# An automated, broad-based, near real-time public health surveillance system using presentations to hospital Emergency Departments in New South Wales, Australia

**DOI:** 10.1186/1471-2458-5-141

**Published:** 2005-12-22

**Authors:** David J Muscatello, Tim Churches, Jill Kaldor, Wei Zheng, Clayton Chiu, Patricia Correll, Louisa Jorm

**Affiliations:** 1 Centre for Epidemiology and Research, New South Wales Department of Health, 73 Miller Street, North Sydney NSW 2059, Australia; 2 Australian Centre for Asthma Monitoring, PO Box M77, Camperdown NSW 2050, Australia

## Abstract

**Background:**

In a climate of concern over bioterrorism threats and emergent diseases, public health authorities are trialling more timely surveillance systems. The 2003 Rugby World Cup (RWC) provided an opportunity to test the viability of a near real-time syndromic surveillance system in metropolitan Sydney, Australia. We describe the development and early results of this largely automated system that used data routinely collected in Emergency Departments (EDs).

**Methods:**

Twelve of 49 EDs in the Sydney metropolitan area automatically transmitted surveillance data from their existing information systems to a central database in near real-time. Information captured for each ED visit included patient demographic details, presenting problem and nursing assessment entered as free-text at triage time, physician-assigned provisional diagnosis codes, and status at departure from the ED. Both diagnoses from the EDs and triage text were used to assign syndrome categories. The text information was automatically classified into one or more of 26 syndrome categories using automated "naïve Bayes" text categorisation techniques. Automated processes were used to analyse both diagnosis and free text-based syndrome data and to produce web-based statistical summaries for daily review. An adjusted cumulative sum (cusum) was used to assess the statistical significance of trends.

**Results:**

During the RWC the system did not identify any major public health threats associated with the tournament, mass gatherings or the influx of visitors. This was consistent with evidence from other sources, although two known outbreaks were already in progress before the tournament. Limited baseline in early monitoring prevented the system from automatically identifying these ongoing outbreaks. Data capture was invisible to clinical staff in EDs and did not add to their workload.

**Conclusion:**

We have demonstrated the feasibility and potential utility of syndromic surveillance using routinely collected data from ED information systems. Key features of our system are its nil impact on clinical staff, and its use of statistical methods to assign syndrome categories based on clinical free text information. The system is ongoing, and has expanded to cover 30 EDs. Results of formal evaluations of both the technical efficiency and the public health impacts of the system will be described subsequently.

## Background

Intentional release of anthrax in the United States in 2001 and the emergence of SARS have highlighted the potential social and economic costs of unanticipated public health threats, and the importance of enhanced preparedness [1-3]. In June 2003, the Centre for Epidemiology and Research (CER) in the New South Wales (NSW) Department of Health, located in Sydney, Australia, embarked on a program to expand and increase the early warning potential of public health surveillance. Additional funding to enhance terrorism preparedness, provided by the NSW Government since July 2003, has been used to cover the establishment and operational costs for the system to date.

The initial focus has been on rapid collection and analysis of information already routinely collected in patient information systems in hospital Emergency Departments (EDs) in NSW. These information systems were initially introduced by the NSW Department of Health in the mid-1990s to help manage ED workloads and to monitor ED performance, but over several years, CER has been exploring secondary use of the resulting data for monitoring syndromic trends. EDs have other appeal for population health surveillance: a large proportion of episodes of medium and high severity acute disease and injury occurring in the community present there on a round-the-clock basis. It is not surprising, therefore, that EDs are central to many public health early warning surveillance systems described in recent years [4-6]. This approach also builds on our previous experience with "drop-in" public health surveillance systems developed for the Sydney 2000 Olympic Games [7].

The Rugby World Cup (the "Cup") is the third largest international sporting event, and it provided an ideal opportunity to trial our proposed ED surveillance system. In 2003, the Cup ran from 10 October to 22 November, giving us only a few months to establish the initial system. Most matches were played in and around metropolitan Sydney, the capital city of NSW. During the months preceding and during the Cup, the number of short-term overseas visitors to Australia increased by approximately 60,000 compared with the same period in the previous year [8].

In 2003, NSW had a population of 6.7 million people, representing one third of Australia's population. More than 60% of the NSW population resides in Sydney, reflecting the high degree of urbanisation [9].

We here describe the methods and review the early results from the new ED-based syndromic surveillance system implemented for the Rugby World Cup period. Our aims were to implement comprehensive, sustainable, and rapid population health surveillance that relied on data collected in ED information systems as part of their routine workflow. We also present the early results from automatic syndrome categorisation that uses presenting problem and triage nurse assessment text to assign syndrome categories.

## Methods

### Consultation and preparation

Most acute care hospitals in Australia are publicly funded and operated, with each State and Territory (and the regional health authorities within them) being responsible for managing local facilities and public health services. Assisted by national and state data standards, there is a reasonable degree of uniformity in the clinical and administrative software deployed in NSW EDs, and in the data collected by them. All public sector EDs in NSW are connected to their hospital computer networks, which are connected to the wide-area networks (WANs) of their regional health authorities, which in turn are connected to the NSW Department of Health state-wide network. A single commercial patient administration and clinical information system, EDIS, provided by HAS Solutions Pty Ltd (now owned by iSOFT Group plc) is used in the majority of NSW EDs [10].

After confirming with its vendor the technical feasibility of adding real-time electronic messaging capabilities to the EDIS software, we consulted with senior ED clinicians and regional health authority managers to gauge reaction to the proposed project and to gather information about any concerns they might have.

Twelve EDs were initially approached on the basis of their proximity to anticipated population and visitor movements during the Rugby World Cup. These EDs represented approximately one quarter of the 49 EDs in the greater Sydney metropolitan region that extends from the Illawarra region to the south of Sydney, through to the Hunter region north of Sydney, and to the Blue Mountains west of Sydney. They account for almost one-third of the approximately 1.2 million ED visits captured annually in electronic ED information systems in NSW.

After modifications to the design of the surveillance system to address resource and governance issues, all EDs agreed to participate. Each ED was provided with supplementary funding to enable the purchase of a desktop computer which could be dedicated to handling the necessary communications tasks, thus avoiding any interference with existing clinical computer workstations. This computer was dubbed the "biosurveillance server" for each ED.

We commissioned the vendor of EDIS to make the necessary changes to its software. These changes are described in the next section. Exhaustive acceptance testing of the software modifications to ensure that they did not compromise normal clinical operations accounted for approximately 40 per cent of the total software modification costs.

### Data capture

Two methods of data capture were implemented. The primary mechanism was real-time HL7 messaging [11]. With this method, each time ED personnel enter or edit any of the EDIS data items that we nominated as surveillance data items, an electronic message in Health Level 7 (HL7) format containing those data items is automatically and immediately forwarded through the NSW Health WAN to a central surveillance database. The specifications of the HL7 message structures are available from the authors on request.

There was some risk that the thorough clinical acceptance testing of the HL7-capable version of EDIS would not be complete in time for the Cup, and we recognised that upgrading EDs to a version of EDIS that had HL7 capacity would take some time. As an interim measure, we asked the software vendor to develop a simple standalone computer program that would extract the nominated surveillance data items into batch files and transfer them through the WAN to the surveillance database using the File Transfer Protocol (FTP). The "Task Scheduler" component of Microsoft Windows was used to automatically initiate this extraction and transmission task every four to six hours in each ED that used this method [12]. Other advantages of this batch extraction program was its capacity to perform, on request, a single large extract of historical data to provide baseline data from each participating ED, and its ability to work with the large variety of EDIS software versions in use at the different EDs.

The forwarded data items comprise the type of visit (such as emergency, planned or unplanned return visit), mode of arrival (such as ambulance or private vehicle), patient age in whole years, sex, country of birth, country of residence (where available), triage nurse-assigned urgency of treatment (triage category), provisional diagnoses (principal and other), and departure disposition (such as discharged, admitted to hospital, transferred or died). The diagnoses are selected by ED clinical staff, mainly by keyword searching to narrow the options and then by selecting the most relevant diagnosis. The EDIS software uses an internal table to translate the chosen diagnoses into codes from the Australian edition of the International Classification of Diseases, version 9, Clinical Modification (ICD-9-CM). Both the code and the text description selected for the diagnosis are included in the HL7 message or batch record which is sent to the surveillance database.

Two additional data items are transmitted: the free-text 'presenting problem' and "nursing assessment" descriptions that are routinely entered by nursing staff during patient triage. The software can also be configured to include the patient medical record number (MRN) in the forwarded data, but this feature can only be enabled by the staff of each ED, not by NSW Department of Health personnel. This ability to forward MRNs will only be used in the event of a declared public health emergency when there is a clear need to facilitate more rapid identification of individual patients requiring further investigation or follow-up. Routinely, neither the MRN nor any other direct patient identifiers, such as name or date of birth, are forwarded to the central surveillance database.

A small suite of computer programs were developed to receive, parse and load the batch files and HL7 messages from the EDs into a central database hosted on computer servers located at the NSW Department of Health data centre. Free, open source software tools, specifically the *PostgreSQL *database and the *Python *programming language, were used for this part of the system [13-15]. The programs written for the NSW system have themselves been released under a free, open source license [16].

In all EDs, batch extraction was used to populate the surveillance database with baseline data starting from 1st September 2003. Subsequent to the Cup, the majority of participating EDs have switched over to using HL7 messaging, although the necessary upgrading and harmonisation of EDIS software versions has taken much longer than originally anticipated.

### Automatic classification of triage text

We used a set of "naïve" Bayesian classifiers to transform free text information on presenting problem and nursing assessment into syndrome categories [17]. This machine learning method has been used in other syndromic surveillance systems to automatically allocate presenting problem text to a small number of categories [18]. The method is termed "naïve" because each word in the text is treated independently of the other words in the text – that is, additional meaning that might be derived from the context of a word within the surrounding text is ignored. Despite this limitation, the method has been used successfully in many applications for automatically classifying documents [19].

We used the *libbow *software library, a sophisticated open source Bayesian classifier package, as the basis of our classification mechanism [20]. TC and JK developed a series of computer programs that incorporated *libbow *software to automatically assemble, process and classify the free text and to assign the relevant categories to ED visit records. The processing steps are explained in more detail below.

Before automatic classification of free text proceeds, all text is automatically "cleaned" in the following phases: conversion of the presenting problem and nursing assessment narrative text to lower case; correction of spelling using context-sensitive fuzzy matching against the Unified Medical Language System (UMLS) lexicon; expansion of common abbreviations using look-up tables, joining of some common phrases into compound words representing a single concept; and "transitive negation" to translate phrases representing negative concepts into compound words representing a single negative concept [21]. For example, "no nausea or vomiting" is transformed into "no-nausea and no-vomiting". Regular expressions are also used to parse quantitative information regarding body temperature, pulse and respiratory rates, and oxygen saturation, and translate these into appropriate categorical descriptions, such as "fever", "no-fever" or "tachycardia".

Bayesian classifiers need to be trained to automatically assign categories. Training requires a dataset that has examples of free text that already have categories assigned. *libbow *then uses the training dataset to learn the probabilities required for applying Bayes' theorem to the pre-processed words in the free text of new ED visits to determine the probability that the text is consistent with a category (the "posterior probability"). Application of Bayes' theorem requires estimation of: the probability that a word occurs in some free text which is representative of a particular syndrome category (known as the "likelihood" of the words); the probability that a category occurs among all ED visits (the "prior probability" of the category) and the probability of each word occurring in all and any ED visit record (prior probability of each of the words). After parsing each parcel of text in the training dataset into a "bag of words" and removing common "stop words" such as "and" and "the", *libbow *software calculates the prior probabilities and likelihoods for each category and for the set of words that occurs in the free text.

To avoid the very large task of manually assigning categories to a sufficient number of ED visits to reliably train the *libbow *classifiers, we elected to use the physician-assigned provisional diagnosis codes in the baseline ED visit data as the categorical basis of the training data set. All baseline data available at the time of training is used in the training. We selected 26 of the 30 syndrome groupings used in our diagnosis-based syndrome reports (described below) to trial the automatic classification algorithm (Table 1). If any ICD-9-CM code for a training visit fell into one of the diagnosis-based syndrome categories, that category was assigned to that training visit.

**Table 1:** Provisional diagnosis and triage text-based syndromes used for statistical reporting and for training the automatic triage text classification system, with correlation between daily counts from the two categorisation methods and the sensitivity of the automatic text classifier in assigning a category that matched the provisional diagnosis category.*

**Syndrome**	**ICD-9-CM codes used for the diagnosis-based syndromes and text classifier training**	**Correlation between daily counts of text and diagnosis-based categories**	**Sensitivity of the text- based category for matching the provisional diagnosis category**
**Selected symptoms**			
Abdominal pain	789.0	0.63	0.94
Chest pain	786.5	0.72	0.92
Convulsions (not clearly epilepsy)	779.0, 780.3	not classified	not classified
Fever (cause unspecified)	780.6	0.54	0.62
Collapse/syncope/coma /delirium/ dizziness	078.81, 780.0, 780.2, 780.4, 992.1	0.64	0.77
Neuromuscular/vision problems	360.8, 368–369, 781–781.99, 782.0	0.57	0.68
**Gastrointestinal syndromes**			
Selected gastrointestinal	001–009, 078.82, 536.2, 558, 564.5, 578.0, 787.0, 787.91	not classified	not classified
Diarrhoea and intestinal infections	001–009, 558, 564.5, 787.91	0.52	0.74
Vomiting	078.82, 536.2, 578.0, 787.0	0.48	0.77
**Cardiovascular syndromes**			
All cardiovascular	390–459, V12.5	not classified	not classified
Cardiac arrest	427.5	not classified	not classified
Cardiac dysrhythmias	427 (excluding 427.5)	not classified	not classified
**Respiratory syndromes**			
All respiratory	033, 460–519, 768–770	0.77	0.74
Asthma	493	0.75	0.81
Influenza	487	0.09	0.22
Pneumonia	480–486	0.38	0.83
Pertussis	033	0.23	0.31
Other/unspecified respiratory infections	460–466 (excluding 466.1), 519.8	0.75	0.79
Respiratory failure/distress	518.5, 518.81–518.82, 519.9, 769	-0.01	0.25
**Injury syndromes**			
All injury and poisoning	800–964.9, 965.09–968.4, 968.6–969.5, 969.8–979, 981–989.4, 989.6–989.9, 990–999 (excluding 992.1)	0.91	0.94
Head injury	800–804, 850–854	0.58	0.59
Burns	940–949	0.78	0.81
Bite or sting (insect/spider/snake)	(910–919).4, (910–919).5, 989.5, E906.2	0.45	0.74
Open wounds	870–897	0.89	0.75
Poisoning (not illicit drug or alcohol)	960–964.9, 965.09–968.4, 968.6–969.5, 969.8–979, 981–989.4, 989.6–989.9	not classified	not classified
**Other syndromes**			
Illicit drug-related (including amphetamines)	292, 304, 305.2–305.9, 965.00–965.02, 968.5, 969.6, 969.7	0.44	0.62
Alcohol-related provisional diagnosis	291, 303, 305.0, 790.3, 980, V70.4	0.78	0.87
Meningococcal infection	036, 320.5	not classified	not classified
Other or unspecified meningitis	047, 320–320.4, 320.6–322	not classified	not classified
Skin problems	035, 050–057, 373, 680–709, 782, 782.1–782.9	0.71	0.85

*Based on all available ED data collected between 1st September 2003 and 29th February 2005. 'Not classified' means that the syndrome categories were not included in the initial implementation of the automated syndrome classification procedure.

To exploit fully the often detailed and multi-faceted triage text that is available, a separate classifier is trained for each category. This means a single ED visit may be assigned multiple syndrome categories. This approach potentially allows monitoring of derived syndrome groupings that represent multiple symptoms occurring together; for example "fever" plus "cough".

Part of the automated training involves determining the maximum number of words (known more generally in machine classification and data mining circles as "features") that are considered meaningful for a category and the threshold posterior probability above which a category is assigned. These values are chosen by our software tools by cycling through permutations of the number of features and probability thresholds and selecting the combination that maximises the Pearson correlation between the daily counts of the baseline computer-assigned categories and the diagnosis-based categories.

Training of the classifier for each syndrome is performed using all available baseline records in the real-time surveillance database that meet the diagnosis-based syndrome criteria at the time the training procedure executes. The text pre-processing steps described above are applied to the historical data prior to training the classifiers. The training proceeds automatically and is repeated at a frequency controlled using configuration options in our own software tools that use *libbow*. This periodic retraining allows the classifier to accommodate changing patterns of word usage. The initial retraining frequency was weekly.

Thus, automated classification of new ED visits that have arrived in the surveillance database proceeds as follows. First the new text is cleaned as described above. For a given category, the probabilities needed to apply Bayes' theorem are calculated for each of the words in the new parcel of text (that is, the new "bag of words"). Bayes' theorem is then used to determine the likelihood that the ED visit belongs to the category given the bag of words. If this probability is above a threshold then the category is assigned to the visit. This process is repeated for each category.

To provide a preliminary assessment of the success of automatic text classification, two methods were used. First, Pearson correlation coefficients were calculated for the association between daily counts of ED visits categorised from provisional diagnoses and from automatic classification of nurse text. Second, the sensitivity was calculated for a text category being assigned. The ED diagnosis-based syndrome was used as a gold standard for the sensitivity calculation. All available ED visits that that had any provisional diagnosis recorded were included in the correlation and sensitivity calculations. The correlations and sensitivity values were updated daily as part of the text classification procedure. While multiple text-based syndromes could be assigned to a visit, one of those syndromes would ideally match the diagnosis-based syndrome. In this preliminary assessment, we did not attempt to assess the accuracy of other text-based syndromes assigned to a visit. While multiple text-based syndromes could be assigned to a visit, one of those syndromes would ideally match the diagnosis-based syndrome. In this preliminary assessment, we did not attempt to assess the accuracy of other text-based syndromes assigned to a visit. However, there should have been sufficient examples of syndromes and diagnoses throughout our database to allow a reasonable assessment of the success of all the text-based syndrome classifiers.

### Statistical analysis and reporting

Cumulative sum ("cusum") techniques have been used in public health surveillance to signal aberrant disease incidence [22]. We developed a modified cusum method for count data to assess the statistical significance and amplitude of increasing incidence of daily ED visits for a syndrome. Using the count on the same weekday of the previous week as the expected count, the method accumulates the sum of the differences between the observed and expected counts. Like other positive cusums, if it falls below zero, it remains at zero, so that only net positive accumulated trends are considered [22,23]. Using the same weekday for comparing counts avoids the problem of comparing two successive days that may be subject to different day-of-week effects. These effects are strongly evident in ED visits and reflect lack of availability of other primary care services after-hours and on weekends, and varying social risk behaviours, such as alcohol consumption on weekends. While this cusum is affected by the variance of both the count in the previous week and the observed count, we elected to use this because we had only one-month of baseline data at the start of the Cup. Despite this additional variability, the accumulated sum of differences will amplify any systematic upward trend in the counts over time.

To make the cusum independent of the level and variability of the background syndrome incidence, two forms of standardisations are applied successively: the first for level and the second for variability. This allows the same interpretation of the degree of increase for different syndromes. The first standardisation is achieved by dividing the accumulated cusum value by the mean syndrome count for the available baseline up to a maximum 365 days. The second standardisation step was introduced because we found the response of this 'mean-standardised' cusum to be sensitive to the variance of the baseline time series; series with high variance were more likely to signal inappropriately than those with low variance. We also found that one-day "differencing" of the strongly autocorrelated mean-standardised cusum produced an approximately normally distributed series. Differencing is commonly used for de-trending time series to allow appropriate parametric analysis of their behaviour [24]. This final standardisation was therefore achieved dividing the mean-standardised cusum by the standard deviation of its differenced values, again using whatever baseline is available up to a maximum of 365 previous days. We called this fully standardised cusum an "index of increase" as it offered a measure of growth in incidence that was comparable across different syndromes, independent of the size and variance of the original counts. Based on empirical testing with many syndrome time series we heuristically selected an index value of ten as a signalling threshold of both statistical and practical significance.

Because of the rapid development required for the Cup and because of the still early developmental nature of the text classification, we presented output from the system in two parallel, automated, daily, prospective reporting streams. Each was available, with password protection, on the NSW Department of Health intranet web site, available for scrutiny by both ED and public health personnel. Reports were refreshed with the latest available data several times each day, to allow trends identified earlier in the day to be re-checked if necessary with more complete data.

First, statistical summaries were prepared for unplanned ED visits grouped into 30 syndromes (Table 1) using the ICD-9 coded ED diagnosis for each visit. These summaries were automatically prepared using scheduled runs of SAS statistical software [25]. Second, for those syndrome categories that were automatically classified from the triage text (Table 1), the open source *Python *programming language and the open source *R *statistical software environment were scheduled to prepare web pages containing graphs of daily counts of each category for the most recent 12 weeks [26,27].

In both types of reports, two levels of ED aggregation were presented for all statistical results. To evaluate geographically localised trends, statistics were available for each ED. To evaluate large-scale trends, the same statistics were presented for the aggregated data for all EDs combined. Web page "hyperlinks" were included in the web reports to navigate among the various reporting levels.

**Figure 1:**
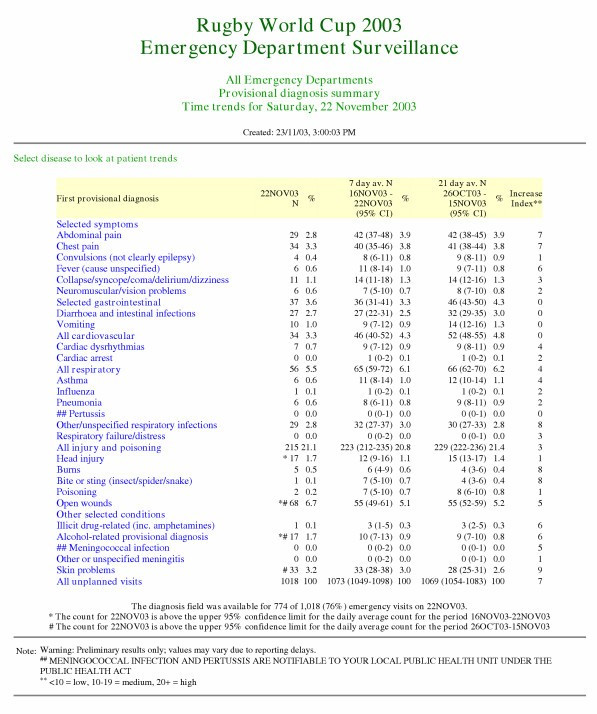
Example of automatically generated intranet report page showing results for the date of the final Rugby World Cup match.

The provisional diagnosis summaries automatically prepared each day included counts of patient presentations for the previous day, the daily average count for the seven days up to and including the previous day, the daily average count for the three weeks prior to that, and the cusum-based index of increase (an example is shown in Figure 1). For each count, 95 per cent confidence intervals were shown, considering each count as the mean of a Poisson distribution [28]. To assist in the assessment of the epidemiological and severity characteristics of any trends, a web page hyperlink associated with each diagnosis category led to an additional web page that summarised the age, triage acuity and admission status of patients in the same three time periods. These summaries showed the proportion of ED visits in each time period for that syndrome that fell into: each of four age categories; a category that combined the most urgent triage acuity categories; and a category representing patients admitted to hospital for further treatment. Each proportion was shown with its 95% binomial confidence intervals.

**Figure 2:**
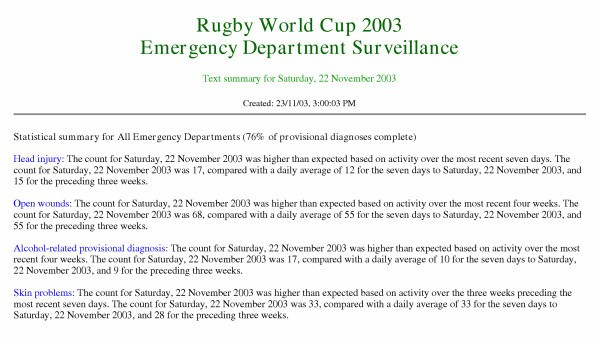
Example of an automatically generated intranet report page showing a sample of the consolidated 'text summary' results for the date of the final Rugby World Cup match.

Each daily statistical summary report provided results for the above statistical calculations for 30 diagnosis-based syndromes for each of 12 EDs, and for all EDs combined; that is, 390 syndrome by ED permutations. To facilitate easier manual review of these reports, any statistically significant result based on the cusum index of increase being greater than ten, or any of the other statistics exceeding the upper value of the one or three-week confidence interval, was automatically listed sequentially in a single web-page called the 'text summary' page. These results were automatically presented as plain English sentences (an example is shown in Figure 2), with the syndrome name created as a hyperlink to the page that summarised the age, triage and admission summary. The text summary was available as a hyperlink from any other web page in the report.

### Public health response

During the Cup tournament, we monitored the automatic reports daily and performed ad hoc analyses to investigate signals. First, any signal was compared against a non-real-time, historical database of ED visits collected by the NSW Department of Health to determine whether a seasonal increase was expected. If an increase was expected then the signal was considered a false alarm. If the surveillance epidemiologist considered that seasonal influences did not adequately explain the increase, then a number of activities were undertaken to evaluate the alarm, including: evaluating the size of the counts that contributed to the signal; the magnitude of the index of increase; the proportions of patients falling within various subgroups based on age, admission status or triage category to determine whether unusual epidemiological features, including changes in the distribution of the severity of cases, were present; descriptive epidemiological analysis to compare the demographic characteristics of the period of increase with a control period, such as a recent period or the same period in previous years from the historical ED database. Finally, if the epidemiologist judged that a concern remained after these analyses, the Rugby World Cup Public Health Committee or other relevant authority, such as the NSW Department of Health Centre for Drug and Alcohol, would be informed.

Because of their early developmental status, trends based on the automatically classified text-based categories were monitored only to assist in qualitatively assessing trends rather than initiating public health action.

The Rugby World Cup Public Health Committee included representatives from Health Protection, Communicable Diseases and Environmental Health Branches of the NSW Department of Health, and Directors of relevant regional Public Health Units. The Committee met on a daily basis, and was responsible for coordinating public health action, and liaising with other agencies such as police and emergency services, if required.

Subsequent to the Rugby World Cup, seven-days-per-week monitoring of surveillance reports by surveillance staff has continued. Signals considered by the surveillance epidemiologist to be unusual in terms of size or non-seasonality are reported to relevant branches of the Department or regional Public Health Units, if initial assessment does not suggest a false alarm.

## Results

### The Rugby World Cup period

At the start of the Cup tournament on 10th October 2003, of the twelve hospitals selected for participation, four were providing data. One of these was providing real-time HL7 messaging and the remainder provided four to six hourly FTP batch feeds. Given the greater complexity of installing HL7 messaging, we elected to install only the simpler FTP batch module at subsequent hospitals during the Cup period, to ensure that we could quickly complete our planned ED participation timetable. Nevertheless, the HL7 site provided data continuously throughout the Cup. By 13th November, all 12 EDs were providing data.

We have records of data completeness for 37 of 44 days (84%) in the Cup period. A median of 93% (range: 58% – 100%) of the ED visits actually occurred were captured in the system by the time the automated report was generated and checked each day. Median completeness of the provisional ICD-coded diagnosis variable was 76% (range: 68% – 86%). While 12 EDs were participating, the median number of ED visits reported per day was 997 (range: 941–1077).

Reports were available for checking on every day during the Cup tournament. Figure 1 shows an example of a report page automatically generated for the final day of the Rugby World Cup (22 November 2003), based on coded diagnosis-based syndromes. Small increases in incidence for that date are evident using the Poisson-based confidence intervals for the "Head injury", "Open wounds", "Alcohol-related provisional diagnosis", and "Skin problems" syndromes. Figure 2 shows the "text summary" representation of these signals. These were not considered sufficient to cause concern, particularly because the Poisson method we used did not adjust for day-of-week effects. The cusum-based index of increase, which does take into account day-of-week influence, supported this conclusion.

The index of increase was incorporated into our automatically generated reports on 29 October 2003. From then to the end of the Cup, we observed 21 signals based on a threshold of ten. This represented an average of 0.8 signals per day, range 0–3 per day. After evaluation, as described in the Methods section above, none of these signals were considered by the surveillance epidemiologist to warrant further action.

Subsequent review of the non-real-time ED database held by the Department revealed a larger-than-usual seasonal increase in diarrhoea-related ED visits in children that had peaked prior to the beginning of the Cup. This coincided with a seasonal increase in rotavirus identification by a major public health laboratory. Examination of the cusum algorithm revealed that the index of increase did not reach a statistically significant value because the diarrhoea-related syndrome counts were already well above background levels when real-time monitoring started, and the available baseline (starting 1 September 2003) was too short to cause the cusum to signal.

The only known outbreak of a reportable disease in progress in urban areas during the months of the Cup was a state-wide outbreak of pertussis [29]. Again, this had commenced prior to the start of the baseline data for the real-time system and as a result the system did not detect an increase in the provisional diagnosis-based pertussis syndrome.

### Automatic classification of nursing text

**Figure 3:**
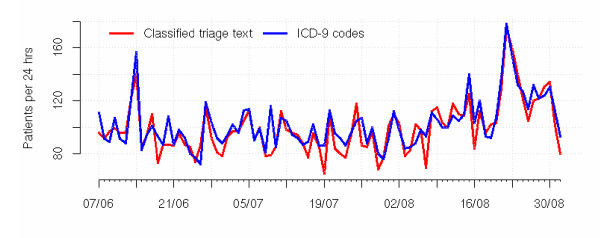
Comparison of daily counts of ED visits for the 'All respiratory' syndrome classified both automatically from triage nurse text and from the coded provisional ED diagnoses, for the period 7 June to 1 September 2004. Includes only ED visits that have a provisional diagnosis recorded.

An example of daily counts of the "all respiratory" syndrome derived from the free text compared with daily counts of the diagnosis-based syndrome is shown in Figure 3. Correlations of daily counts of the two syndrome categorisation methods varied between syndromes from very strong to very weak. "All injury and poisoning", "open wounds", "burns", "alcohol-related provisional diagnosis", "all respiratory", "asthma", "other/unspecified respiratory infections", "chest pain", and "skin problems" all had correlation coefficients above 0.7. "Pertussis", "influenza", and "respiratory failure/distress" had coefficients below 0.3 (Table 1). The sensitivity of the automatic classifier for assigning a category that matched a provisional diagnosis category of an ED visit also varied from very strong to poor. "Abdominal pain", "all injury and poisoning" and "Chest pain" had sensitivities of more than 90%. "Respiratory failure/distress" and "influenza", had sensitivities below 30% (Table 1).

## Discussion

In just over three months from the commencement of funding, we implemented an automated syndromic surveillance system of broad scope that took maximal advantage of existing information systems and communications infrastructure. The system was designed to have negligible impact on the operation of participating EDs and this was reflected in the completeness of ED data available for reporting on a daily basis during the Rugby World Cup. More than 90% of ED visits, on average, were available at the time we checked the automatically generated reports each day.

Unlike the ED-based public health surveillance system which was established for the Sydney 2000 Olympic Games, we believe our current ED-based system to be highly sustainable [7]. The Olympic system required additional staff to perform manual data abstraction and data entry, and was both expensive to operate and somewhat disruptive to normal ED operations.

While current reporting is up to midnight on the previous day, near instantaneous HL7 messaging and/or frequent automated batch feeds give us the future flexibility to report on trends within recent hours rather than the most recent day. We continue to expand the use of HL7 messaging by participating EDs. Our experience is that this provides a robust method of delivery and requires less maintenance than the FTP batch method. However, barriers to greater implementation of HL7 messaging include the need for some HL7 expertise amongst local information system support staff at participating sites, the variety of EDIS software versions from the same vendor that are implemented at different EDs (only recent versions have had HL7 capacity added to them), and the need for HL7 message routing infrastructure within the regional WAN. The FTP batch method, because it only relies on a standalone software module that communicates directly with our data centre, is much less dependent on the currency of EDIS software versions in use in each ED.

Novel aspects of our syndrome classification system include the availability of both ICD-coded ED diagnoses and free text for classifying syndromes, the use of more detailed nursing assessment text as well as terse presenting problem text in the syndrome classification procedure, and the use of the ICD-coded diagnoses to train (and periodically re-train) the automatic syndrome classifier. Diagnosis and free text-based syndromes each have advantages and disadvantages for use in syndromic surveillance. While the diagnoses can be more specific than automatically classified syndromes, they are entered by busy ED staff who are not trained in medical coding and therefore their accuracy can vary. Secondly, diagnoses are not entered until the end of the visit, or some time thereafter, and are sometimes not entered at all, as evidenced by the one quarter of ED visits that did not have a diagnosis at the time our daily reports were checked during the Rugby World Cup. The triage text is routinely entered in the ED information system shortly after the patient's arrival at the ED and is therefore available for syndrome classification as soon as it arrives in our database. Thirdly, depending on the level of diagnostic certainty available at the end of the ED visit, the diagnosis entered may reflect a symptom or a specific diagnosis, but not both. For example, a patient presenting with chest pain, who is later diagnosed with a cardiac event, may be assigned a principal diagnosis code that represents only chest pain, or a diagnosis code that represents the cardiac event. This is an additional, unwanted source of variability. Fourthly, a mean of only 1.1 diagnosis codes are entered in the ED information system for each patient presentation to an ED. Thus, only a limited part, if any, of the presenting syndrome may be reflected in the ICD codes that are recorded. In contrast, the free text classification algorithm can assign as many categories as are warranted by the textual description, thereby potentially offering a fuller picture of the presenting syndrome. Finally, new text-based syndrome categories can be introduced into the system and retrospectively assigned to ED visits as required, simply by training a classifier and reprocessing historical text information through it.

Based on this preliminary evaluation, automatic classification of triage nurse text into syndromes worked well for many symptoms and syndromes, particularly those with large counts and broad meaning, such as injury and respiratory disease. Specific diagnoses such as pertussis and pneumonia showed poor results, probably because it is unrealistic to expect the classifier to identify specific diagnoses given only the information available at triage. This confirms that automated text classification is best used for syndromes that are symptom rather than diagnosis-based. Other categories, particularly those with small counts such as "respiratory distress", were not classified well. More formal evaluation is required, one that uses a better gold standard than the provisional diagnoses, such as systematic classification by humans who are blinded to the final diagnosis. To balance the limitations of each method of syndrome classification, we continue to use both methods in our ongoing system.

The cusum-based index of increase which we developed appears to provide a more useful and easily interpretable assessment of a sustained increase in incidence than does the simple Poisson count method we used. We find that a rising value of the index will continue to demand the attention of surveillance personnel because this means incidence is continuing to rise. The Poisson method was oversensitive and appeared to trigger false alarms simply because of day-of-week effects and because of chance increases that are inevitable when making so many multiple comparisons. Because we are monitoring many health categories, it was important to have a measure that did not require a different interpretation for each category. There are other advantages to the cusum approach: its use of the previous weekday count instead of a longer term average for the expected value means it only needs a short baseline to become useful, as long as the baseline time series is operating under non-epidemic conditions. Shorter baseline methods have been shown to be as effective as those requiring several years of baseline [30]. However, the missed diarrhoeal and pertussis outbreaks before and during the Cup highlight the risk of relying on too short a baseline in the early life of a system such as this. Unlike the traditional cusum, our modified cusum quickly returns to baseline levels when the underlying process does, without needing to be reset. Its value provides a threshold for triggering, but after it triggers it can be monitored to assess the relative and ongoing scale of continued increase in incidence. Its primary disadvantage is that it does not adjust for annual seasonal trends. We therefore have to manually examine historical trends to assess whether seasonal increases are progressing beyond seasonal levels. In some cases this is not a disadvantage. We believe it useful to trigger an alert at the start of the annual influenza season, for example, even though it occurs every year. The impact of the circulating influenza on ED visits as marked by the index of increase may provide a useful assessment of the virulence or degree of transmission of the circulating influenza strain, although this needs further evaluation. A related disadvantage is that a sustained seasonal increase will affect the 365-day mean used in the cusum's adjustment, making it less sensitive to subsequent increase in syndrome incidence. However, the moving 365-day mean changes more slowly than the short-term increase that the cusum is designed to detect, making this less of a problem. The choice of a maximum 365-day baseline was a balance between preventing the standardised cusum from being unduly influenced by long-term secular trends while not being too sensitive to short-term trends. The cusum continues to provide a useful signalling tool while we explore more sophisticated statistical methods.

Unlike other ED-based systems, we have not yet put a strong emphasis on geographically based statistical aberration techniques [5,6,31]. For simplicity, we have elected to focus on the ED as a marker of geographic location rather than the patient's residential address, although we will be trying alternatives as further development of the system progresses. While the address of the ED attended is a rather coarse measure of the relevant geographic location, home address may also be of limited value because exposure to a biological agent could occur at work, at school or elsewhere in the community.

While the system has since proven useful for a range of public health activities and problems as diverse as influenza surveillance, short-term increases in acute asthma incidence in children and in recreational drug misuse, more formal, objective evaluation of its likely performance in recognising the disasters that it is primarily designed to prevent is required. In relation to bioterrorism, challenges include developing more specific indicators for potential scenarios and ensuring that all important symptom groups are covered. Development of methods to extract information about external causes of injury from the free text is underway, and may offer benefits for injury prevention. A comprehensive indexed word (and partial word or wildcard) search facility has also been added to facilitate querying the triage nurse text, to allow ad hoc searches for specific types of presentation. This has proven to be useful, for example, in determining how frequently a particular illicit drug is mentioned in the triage text. We are also actively working with partner organizations on the development of several statistical model-based approaches to outbreak and anomaly detection, in the hope of improving upon the performance of the simple modified cusum which we are currently using. We see the refinement of practical and reliable detection algorithms and models which can be used prospectively, in near real-time, without the need for constant manual adjustment or optimisation, as the most pressing and difficult challenge for near real-time public health surveillance systems.

Public health services are also challenged to develop appropriate responses to the information generated by new, non-traditional public health surveillance systems such as ours. As we have demonstrated, it is relatively easy to establish a system which can collect large volumes of public health surveillance data with very little delay. It is much more difficult to determine, on a continuous basis, whether that data contains information which demands public health action, or more likely, warrants further investigation. Near real-time information demands a near real-time response, which may be difficult to achieve with existing staffing levels and work practices. Such systems also potentially generate information on issues that may not have previously been the subject of rapid, organised public health responses, such as clusters of presentations related to illicit drugs, specific causes of injuries or asthma.

Furthermore, identifying and ascribing syndrome trends to specific causes or organisms remains a challenge that is the subject of ongoing evaluation and development. It is difficult to mount a public health response in response to an increase in the incidence of a symptom that in most cases represents a naturally occurring, self-limiting illness. Identifying that the observed increase relates to the early activity of an introduced bioterrorism agent requires extremely careful judgement on the part of surveillance system staff and extensive knowledge of the epidemiology of the non-specific syndromes they are monitoring. For this reason, in judging the importance of any unusual syndrome activity, we rely heavily on variables from the ED information system that mark the severity of the presenting syndrome, as well as the scale of an apparent increase in syndrome activity.

## Conclusion

Through secondary use of routinely collected ED information system data, a high degree of automation, and thoughtful use of readily available web-based reporting technology, we developed a public health surveillance system that provides a much more timely, broad-based surveillance capability than was previously available in NSW. This does not replace notifiable communicable disease surveillance, but complements its greater specificity but narrower scope. As the SARS outbreaks in China, Hong Kong, Singapore and Toronto have illustrated, the social and economic impact of public health disasters can be enormous. Early identification of an emerging problem is crucial to mitigating the impact of a disaster. The role of this system in such public health disasters needs further evaluation. In the intervals between disasters, these systems need to maintain continuing relevance by rapidly providing useful information on the ubiquitous public health problems which health departments must address every day.

## Competing interests

The author(s) declare that they have no competing interests.

## Authors' contributions

All of the authors participated in the establishment of the system, and all read and approved the manuscript. TC, JK, PC and CC developed the text processing and classification system. TC, WZ and DM developed and tested the central database and the reporting systems. DM developed the modified cusum statistic. DM, WZ and TC are responsible for day-to-day operation of the system. DM, CC and TC wrote the initial draft of the manuscript, which TC and LJ edited.

## Pre-publication history

The pre-publication history for this paper can be accessed here:



## References

[B1] United States General Accounting Office (2003). Bioterrorism: Public health response to anthrax incidents of 2001 GAO-04-152 Washington.

[B2] Kaufmann AF, Meltzer MI, Schmid GP (1997). The economic impact of a bioterrorist attack: Are prevention and postattack intervention programs justifiable?. Emerg Infect Dis.

[B3] Campbell A (2004). The SARS Commission Interim Report: SARS and public health in Ontario.

[B4] Gesteland PH, Gardner RM, Tsui FC, Espino JU, Rolfs RT, James BC, Chapman WW, Moore AW, Wagner MM (2003). Automated syndromic surveillance for the 2002 Winter Olympics. JAMIA.

[B5] Heffernan R, Mostashari F, Das D, Karpati A, Kulldorff M, Weiss D (2004). Syndromic Surveillance in Public Health Practice, New York City. Emerg Infect Dis.

[B6] Lombardo J, Burkom H, Elbert E, Magruder S, Lewis SH, Loschen W, Sari J, Sniegoski C, Wojcik R, Pavlin J (2003). A systems overview of the Electronic Surveillance System for the Early Notification of Community-Based Epidemics (ESSENCE II). J Urban Health.

[B7] Jorm LR, Thackway SV, Churches TR, Hills MW (2003). Watching the Games: public health surveillance for the Sydney 2000 Olympic Games. J Epidemiol Community Health.

[B8] Australian Bureau of Statistics (2005). Short-term visitor arrivals to Australia, Preliminary (catalogue 3401055001).

[B9] Australian Bureau of Statistics (2005). New South Wales in Focus (catalogue 13381).

[B10] HAS Solutions Pty Ltd (2003). Emergency Department Information System (EDIS), versions 6 and 9 [computer program].

[B11] Health Level Seven Australia Health Level Seven V2.3.1. http://www.hl7.org.au/HL7-V2-Resrcs.htm.

[B12] Microsoft Corporation (2003). Microsoft Windows [computer program].

[B13] Open Source Initiative Board of Directors Open Source Initiative. http://www.opensource.org.

[B14] PostgreSQL [computer program].

[B15] Python [computer programming language].

[B16] Centre for Epidemiology and Research NSW Department of Health Open source tools for population health epidemiology. http://www.health.nsw.gov.au/public-health/epi/open_source_tools.html.

[B17] Mitchell T (1997). Machine Learning.

[B18] Espino JU, Wagner M, Szczepaniak C, Tsui FC, Su H, Olszewski R, Liu Z, Chapman W, Zeng X, Ma L, Lu Z, Dara J (2004). Removing a barrier to computer-based outbreak and disease surveillance – the RODS Open Source Project. MMWR Morb Mortal Wkly Rep.

[B19] Lewis DD (1998). Naive (Bayes) at forty: The independence assumption in information retrieval. ECML'98, The Tenth European Conference on Machine Learning.

[B20] McCallum AK Bow: A toolkit for statistical language modeling, text retrieval, classification and clustering. http://www.cs.cmu.edu/~mccallum/bow.

[B21] Browne AC, Divita G, Aronson AR, McCray AT (2003). UMLS language and vocabulary tools. AMIA Annu Symp Proc.

[B22] Hutwagner LC, Maloney EK, Bean NH, Slutsker L, Martin SM (1997). Using laboratory-based surveillance data for prevention: an algorithm for detecting Salmonella outbreaks. Emerg Infect Dis.

[B23] Lucas JM (1985). Counted data cusums. Technometrics.

[B24] Chatfield C (2004). The analysis of time series: an introduction.

[B25] (2001). SAS version 8 [computer program].

[B26] van Rossum G, Drake FL, eds Python Reference Manual.

[B27] R Development Core Team R: A language and environment for statistical computing.

[B28] Armitage P, Berry G, Matthews JNS (2002). Statistical methods in medical research.

[B29] Communicable Diseases Branch NSW Department of Health (2004). Communicable diseases report, NSW, for October and November 2003. NSW Public Health Bull.

[B30] Hutwagner L, Browne T, Seeman GM, Fleischauer AT (2005). Comparing aberration detection methods with simulated data. Emerg Infect Dis.

[B31] Olson KL, Bonetti M, Pagano M, Mandl KD (2005). Real time spatial cluster detection using interpoint distances among precise patient locations. BMC Med Inform Decis Mak.

